# Crosstalk Between Oral Microbiome and Cancer: Emerging Trends and Insights

**DOI:** 10.1155/cjid/6639127

**Published:** 2025-09-20

**Authors:** Shanshan Yang, Shaodong Hao, Hui Ye, Xuezhi Zhang

**Affiliations:** ^1^Traditional Chinese Medicine and Integrated Medicine Department, Peking University First Hospital, Beijing, China; ^2^Spleen-Stomach Department, Fangshan Hospital, Beijing University of Chinese Medicine, Beijing, China

**Keywords:** cancer, high-cited papers, hotspots, oral microbiome, visual analysis

## Abstract

**Background:** Emerging scientific evidence suggests a connection between the oral microbiome (OM) and cancer (CA). This paper is designed to delve into the scientific output within this domain, pinpoint highly cited articles, and explore the latest research hotspots and emerging trends in OM/CA studies.

**Methods:** On January 25, 2025, a comprehensive search was conducted to investigate research on the relationship between OM and CA. The search terms pertinent to both OM and CA were utilized. Key information was extracted from WoSCC. A suite of tools including Biblioshiny from R packages, Excel, and VOSviewer were utilized for comprehensive data visualization. Co-citation analysis was conducted to delineate the conceptual landscape of the field and to highlight influential publications.

**Results:** A total of 1663 papers related to OM/CA were retrieved. The number of papers (Np) in OM/CA is proliferated from 2010 to 2024. The United States and China occupied leading positions and made the most significant contributions. Karolinska Institutet and Harvard University were the most productive institutions. The most prolific authors were Tina Dalianis and Anders Naesman. Oral Oncology garnered the most Np, whereas Gut received the highest total citations (TCs). A historical citation analysis traced the evolution of OM/CA research over time. The key topics encompassed the impact of OM on the initiation and progression of CA, the characteristics of OM in CA, the role of OM in screening and prognosis of CA, the effect of OM on CA treatment, and the underlying mechanisms through which OM is involved in CA. Emerging research hotspots in this field may include Mendelian randomization, applications of machine learning, biofilm formation, cytolethal distending toxin, and nanoparticles, along with the roles of *Fusobacterium nucleatum* and *Porphyromonas gingivalis* in disease pathogenesis.

**Conclusion:** This study assessed worldwide production in OM/CA research, examining its quantitative characteristics. It pinpointed several pivotal papers and compiled data on the current status, emerging hotspots, and evolving trends within OM/CA research. The findings of this research may offer a fresh perspective for both academics and practitioners in the field.

## 1. Introduction

Despite significant advancements achieved over the past century, cancer (CA) continues to pose a major concern for global public health [1]. Early detection and intervention are paramount in the management of CA. The oral microbiome (OM), a highly intricate ecosystem harboring approximately 1000 microbial species alongside diverse viruses, fungi, and protozoa, represents the human body's second most complex microbiome after the gut [2, 3]. Defining this dynamic community and its dysbiosis is essential in CA management [4, 5]. Recent revolutions in sequencing, mass spectrometry, third-generation Oxford Nanopore Technology (ONT), bioinformatics, and machine learning (ML) enable unprecedented resolution analysis. State-of-the-art omics tools including genomics, metagenomics, phylogenomics, pangenomics, transcriptomics, proteomics, metabolomics, lipidomics, and integrated multiomics now empower comprehensive characterization of OM structure and function, advancing our understanding of OM and disease mechanisms at unprecedented depth and scale [3, 6, 7].

In recent years, OM has garnered attention as a potential causative agent for numerous diseases [2, 8]. Consequently, the relationship between OM and CA has emerged as a focal point of intense research [4, 5, 9]. Numerous studies show OM affects CA initiation, progression, invasion, and metastasis via mechanisms like modulating inflammation and immune responses, inducing DNA damage and cellular proliferation, and producing carcinogens [10, 11]. OM can be used as biomarkers for the detection of CA, such as oral cancer [12], esophageal cancer [13], gastric cancer (GC) [14], pancreatic cancer [15], and colorectal cancer (CRC) [16]. Moreover, OM can modify the outcomes of CA treatment and be utilized to predict the prognosis of CA [17]. Modulating OM may be an approach for CA management [18, 19].

Bibliometrics facilitates the visualization of trending topics and the evolution of knowledge within specific research domains, achieving notable success in medical studies. Various related topics on the correlation between gut microbiome (GM) and CA have been extensively explored through bibliometric analysis, including the links between GM and CA [20], the interaction between GM and liver cancer [21], and the interplay between GM and CRC [22]. Over the past decade, there has been a consistent rise in the number of clinical and animal studies investigating the connections between OM and CA. Nevertheless, to date, no research has been published on the quantitative examination of the links between OM and CA. This paper endeavors to identify papers pertinent to OM/CA over the past 15 years, dissects their characteristics, and conducts a visual analysis and knowledge mapping of bibliometric indicators such as research hotspots, topics, and publishing status within the OM/CA field to aid scholars in better understanding the dynamic shifts and development trends in OM/CA research.

## 2. Materials and Methods

### 2.1. Data Source and Search Strategy

Different databases have varying selection criteria and indexing methods, leading to potential inconsistencies in data quality and comparability. This bibliometrics primarily utilizes the Web of Science Core Collection (WoSCC) database, which is a prestigious academic database widely employed in the scientific research community and ensures comprehensive coverage of authoritative and influential journals across various disciplines. Additionally, WoSCC allows for the filtering of search results based on publication year and document type, facilitating a refined and targeted analysis of the scholarly landscape.

**Figure 1:**
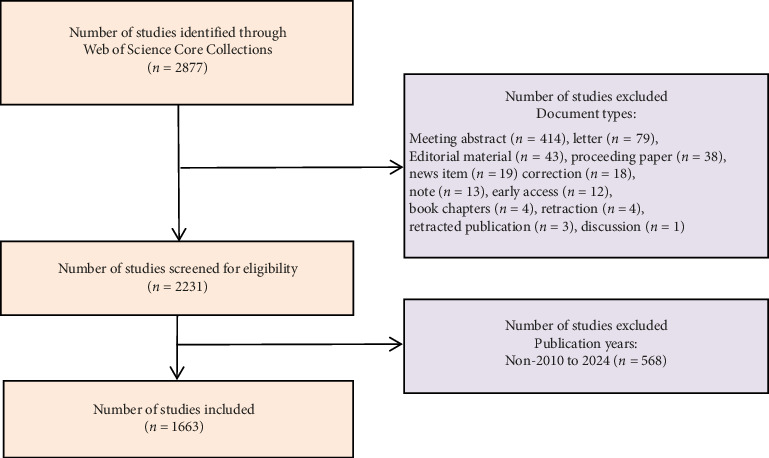
Flowchart of literature screening in OM/CA.

All search queries were executed and retrieved from the WoSCC database on January 25, 2025 (Eastern Eighth Time Zone, ET8). A meticulously curated set of keywords pertaining to “oral microbiome” and “cancer” were utilized to identify pertinent articles. These search terms, along with their synonyms, were meticulously formulated using the “advanced search” methodology. The comprehensive search strategy is detailed in Supporting Information. Inclusion criteria were as follows: (1) Publications dated between January 1, 2010, and December 31, 2024. (2) Document types limited to original research articles and reviews. (3) No language restrictions applied. Exclusion criteria were as follows: (1) Publications outside the specified date range (pre-2010 or post-2024). (2) Publication types include meeting abstracts, letters, editorials, corrections, news items, proceedings papers, book chapters, and retracted publications. Consequently, a comprehensive total of 1663 papers were retrieved (Figure 1), comprising 1366 articles and 297 reviews. The search and subsequent data extraction were meticulously conducted by two researchers, SY and SH. From the raw data obtained, we extracted pertinent information and stored it in a text format for subsequent refinement and analysis.

### 2.2. Data Analysis and Parameter Query

A scientometric analysis was conducted using a combination of tools, including Biblioshiny from the Bibliometrix R package (Version 4.1.3, originating from Boston, MA, USA), VOSviewer (Version 1.6.18, developed by Leiden University in the Netherlands), a data visualization platform accessible at https://www.chiplot.online/, and Microsoft Excel 2019 (Microsoft, Washington, USA). The Bibliometrix (https://www.bibliometrix.org/) offers a suite of tools for scientometric analysis, while VOSviewer serves as a powerful platform for creating and visualizing bibliometric networks.

The analysis comprehensively examined various facets of scientific production, encompassing pertinent journals, authors, affiliations, and countries. Additionally, it delved into various citation metrics, such as the H-index for local impact, total citations (TCs), and annual production trends over time. The H-index is the highest rank *h* such that the researcher has at least *h* publications each cited at least *h* times. TCs are used to measure the number of times a paper is cited. High citation frequency usually implies that the article has great influence. These metrics provided insights into the evolving landscape of scientific research. Furthermore, the study extended its focus to investigate main funding agencies, country scientific output, and country collaboration networks. The analysis also explored historical direct citation networks, highly cited papers, and high-impact factor (IF) papers. These elements were crucial in understanding the influence and impact of specific research papers within their respective fields. The results were vividly presented through graphs, charts, and network maps. These visualizations not only facilitated easy understanding of the data but also allowed for deeper insights into the patterns and trends observed in scientific production. The journal's JCR Quartile and IF were defined by the “2023 Incites Journal Citation Report,” a reliable source for evaluating the journal's performance and impact.

## 3. Results

### 3.1. Annual Publication Outputs

**Figure 2:**
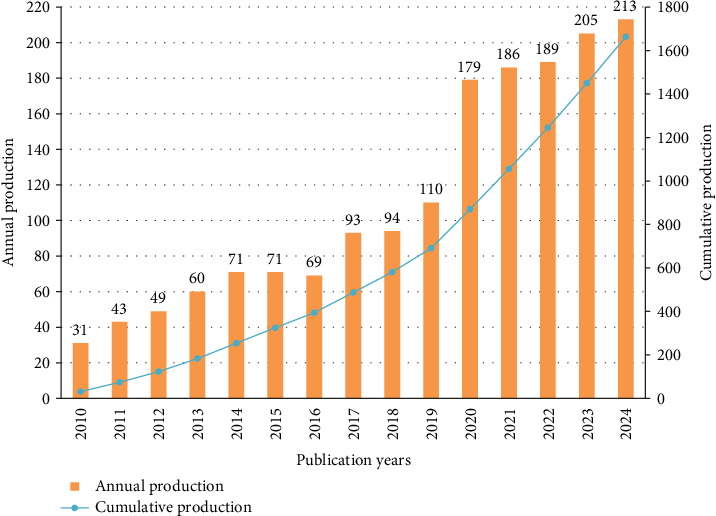
Annual and cumulative scientific output in OM/CA.

Annual scientific output serves as a crucial indicator, effectively reflecting the prevalent research trend in a particular field. The Np in this specific domain serves as a reflective indicator of its evolutionary trajectory.  Figure 2 depicts the annual and cumulative Np within OM/CA research spanning from 2010 to 2024, revealing a notable upward trend in overall Np. Notably, the period between 2018 and 2020 witnessed a particularly striking surge in Np, indicating a substantial growth in research output. Furthermore, from 2021 to 2024, the Np exceeded 180 annually. Overall, the surge in the Np during this period could be intricately tied to the broadening scope of research fields, the increase in scientific research investment, or the frequent academic exchange activities.

### 3.2. Main Journals

**Figure 3:**
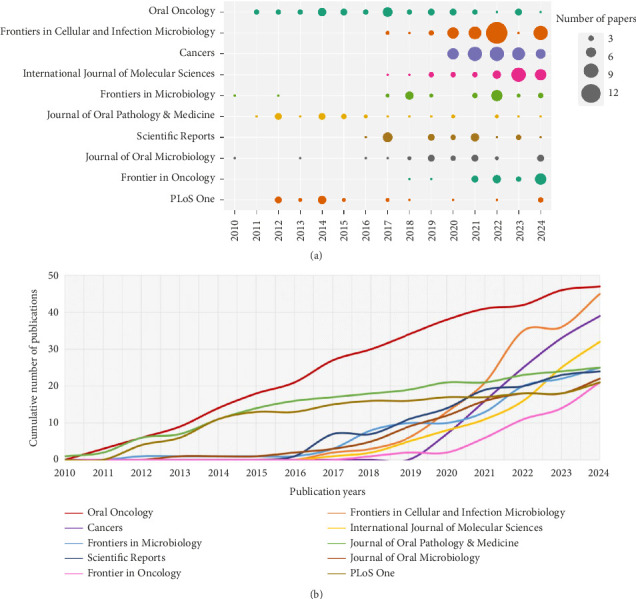
(a) Annual production over time for the top 10 most productive journals in OM/CA (the size of the circle corresponds to the number of papers, with a larger circle signifying a higher production). (b) Cumulative output of the top 10 most productive journals in OM/CA.

Table 1 presents the top 10 most productive journals, with Oral Oncology topping the list with the highest Np (*n* = 47), closely followed by Frontiers in Cellular and Infection Microbiology (*n* = 45), *Cancers* (*n* = 39), and International Journal of Molecular Sciences (*n* = 32). TC serves as an indicator of the significance of these journals, while the H-index provides a measure of their academic impact. Oral Oncology had the highest citations and H-index among high-yield journals.  Figure 3(a) illustrates the annual Np of the top 10 journals, revealing that Frontiers in Cellular and Infection Microbiology emerged as the most productive journal in 2024.  Figure 3(b) summarizes the cumulative Np of these top 10 journals, which amounted to a total of 301 publications, accounting for approximately 18.10% of the overall output. This highlights the exceptional production capacity of these journals.

### 3.3. Main Authors and Institutions

**Figure 4:**
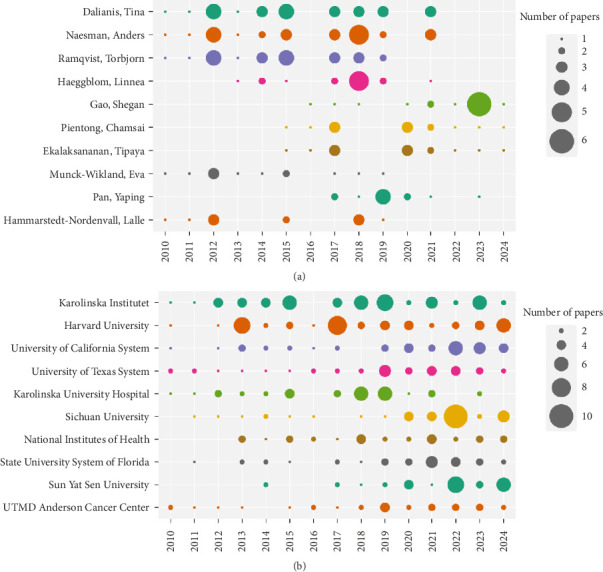
(a) Annual output over time for the top 10 most productive authors in OM/CA (circle size indicates scientific output, with larger circles representing greater output). (b) Annual output of the top 10 institutions overtime in OM/CA.

Table 2 lists the top 10 most productive authors, including their TCs and H-index, with Dalianis Tina, Naesman Anders, Ramqvist Torbjorn, Haeggblom Linnea, and Gao Shegan ranking among the top five. Half of the top 10 authors were from Karolinska Institutet and Karolinska University Hospital. Notably, Dalianis Tina held the distinction of having the highest Np, the highest H-index, and the highest TC in OM/CA research, indicating her significant contribution.  Figure 4(a) illustrates the annual output of these top 10 authors, revealing that most of their papers were published in 2017.

Table 3 presents the top 10 most productive institutions, with Karolinska Institutet from Sweden boasting the highest Np. However, Harvard University from the United States took the lead in terms of the highest TC and H-index. The University of California System and the University of Texas System also made significant contributions. In China, Sichuan University and Sun Yat Sen University stood out as the major contributors to the articles. Among the top 10 institutions, six were from the United States, two from Sweden, and two from China.  Figure 4(b) displays the annual Np of these top 10 institutions between 2010 and 2024, providing a comprehensive overview of their productivity trends over the years.

### 3.4. Main Countries/Regions and Funding Agencies

**Figure 5:**
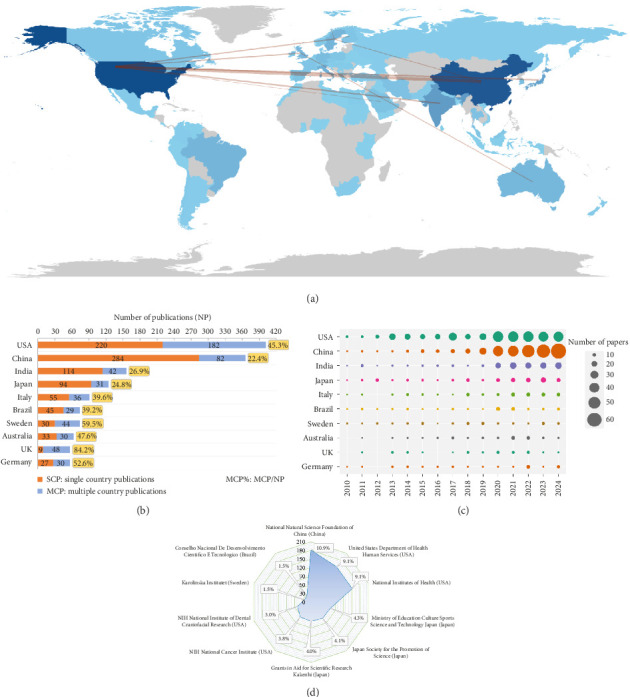
(a) Scientific productivity and main collaborative dynamics among countries/regions in the field of OM/CA (depicted through a color palette combining blue and gray hues: gray signifies countries/regions devoid of any published documents, while varying shades of blue reflect the volume of publications, with lighter shades indicating fewer and darker shades signifying a greater number of documents; red lines illustrate the interconnections between countries, with thicker lines depicting stronger ties and thinner lines weaker ones). (b) International cooperation of publications in the top 10 high-yield countries in OM/CA research (orange SCP denotes the count of papers authored solely by individuals from a single country, whereas blue MCP represents the count of papers authored by individuals from multiple countries; the MCP ratio serves as an indicator of the level of international cooperation). (c) Annual output of the top 10 productive countries over time in OM/CA. (d) The top 10 funding agencies and source countries in OM/CA.

Table 4 presents the top 10 countries based on the quantity of publications, clearly indicating that the United States (*n* = 402) and China (*n* = 366) were the primary sources of papers, collectively accounting for approximately 46.18% of the total scientific output. The United States led with 402 papers, 15,113 citations, and an H-index of 67. China, which held the second-highest publication volume of 366, also secured the second spot in both TC and H-index. This may be related to the high attention and support of the two countries on the OM/CA program.  Figure 5(a) provides a vivid illustration of the scientific contributions from various countries and the intricate collaboration network among them. Notably, the United States stands out as a leading figure in international cooperation, with China being its closest and most frequent collaborator. Additionally, Figure 5(b) offers insights into the extent of collaboration of authors from top 10 productive countries, highlighting an intriguing trend of prevalent international cooperation in Sweden, the UK, and Germany. Meanwhile, Figure 5(c) depicts the annual Np for the top 10 countries, revealing a significant shift in scientific productivity. Prior to 2020, the United States held the top position, but since then, China's annual Np had surpassed that of the United States. Lastly, Figure 5(d) outlines the key funding agencies that support OM/CA research, predominantly from China, the USA, and Japan, indicating their strong commitment and substantial support for advancing research in this field.

### 3.5. Analysis of Cited Papers in OM/CA Research

#### 3.5.1. High-Cited Articles in OM/CA Research Based on WoSCC

Table 5 presents the top 20 high-cited papers in original OM/CA research. These articles were mainly from the JCR Q1 partition, the time span was from 2010 to 2018, the TCs were more than 150, and the Gut had published the most influential papers. We summarized the following three points.

First, there were distinguished differences in the structure of OM between CA patients and healthy controls (HCs). Two articles [23, 24] published in Gut found the potential association between OM variation and pancreatic cancer. Based on the differences in OM between healthy and diseased individuals, OM could be used for CA screening. OM composition might reflect the risk of oral cancer [25–28], esophageal cancer [13], and CRC [18]. Increased antibody levels against specific symbiotic oral bacteria could inhibit the growth of pathogenic bacteria and reduce the risk of pancreatic cancer [29]. The mortality of oral and digestive tract cancer may be related to the *Porphyromonas gingivalis* (*P. gingivalis*) serum antibody levels [30]. On the other hand, there might be key differences in the structure of OM between tumor tissues and nontumor tissues. Potential changes in bacterial diversity in nontumor and tumor mucosal tissues of OSCC indicated a shift in bacterial colonization [31].

Second, special OM may play a significant role in CA. Specific OM may promote the onset and development of tumors. For example, periodontal pathogens *P. gingivalis* and *Fusobacterium nucleatum* (*F*. *nucleatum*) could promote the progression of oral squamous cell carcinoma (OSCC) [32]. *P. gingivalis* infection of esophageal epithelium in esophageal squamous cell carcinoma (ESCC) patients may be positively correlated with the differentiation status, metastasis, and overall survival of ESCC [33]. *P. gingivalis* could promote the invasion of OSCC by inducing proMMP9 and its activation [34]. In addition, *human papillomavirus* (HPV) may play a key role in the occurrence and development of OSCC [35] and head and neck cancer (HNC) [36] and may also increase the incidence of base of tongue cancer [37].

Third, OM intervention may be used as CA treatment [26]. For instance, the higher oral abundance of symbiotic *Corynebacterium* and *Kingella* might be associated with a reduced risk of head and neck squamous cell carcinoma (HNSCC), which has potential significance for CA prevention [9]. A study [38] reported a new strategy to engineer cationic nanoparticle-coated bacterial vectors that could efficiently deliver oral DNA vaccine for contaminated CA immunotherapy. Oral photothermal bacteria that regulate TNF-α expression could be used to mediate tumor therapy [39].

#### 3.5.2. High-Cited Reviews in OM/CA Research Based on WoSCC

A review offers timely and invaluable guidance to scholars, encapsulating a vast array of information that encompasses recent advancements in research, prevalent issues within the discipline, and anticipated future trends.  Table 6 shows the top 10 most cited reviews. It is evident that most of the highly cited reviews are not systematic reviews and meta-analyses but narrative reviews. Several reviews [4, 7, 40–43] describe the possible role of OM in tumorigenesis and CA development, especially oral cancer and CRC. A review [28] summarizes the role of oral microbial characteristics in tumor diagnosis and prognosis. Moreover, OM may be a potential target for CA treatment [4]. The OM–GM crosstalk might play a significant yet underappreciated role in the pathogenesis of gastrointestinal cancer, influencing diagnosis, prognosis, and treatment [44]. A systematic review [45] suggests a potentially important causal association between HPV and oral carcinoma and oral potentially malignant disorders (OPMD). A review [46] outlined the interactions between *P. gingivalis* or *F. nucleatum* and epithelial cells that could produce an oncogenic phenotype, and *P. gingivalis* or *F. nucleatum* could be used as a poor prognostic indicator.

#### 3.5.3. Historical Cited Papers in OM/CA Research Based on WoSCC

**Figure 6:**
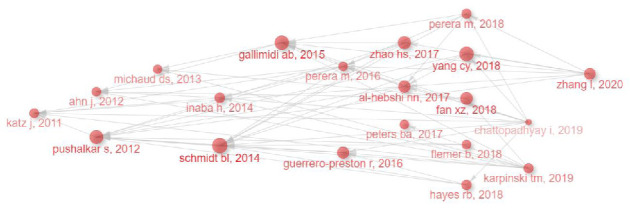
Historical citation network in OM/CA (gray lines symbolizing citation relationships, while each dot on the network represents an individual paper, marked by its author and publication year).

Through a historiographical analysis using Bibliometrix (Figure 6), several seminal papers in OM/CA research were identified. Their local citation scores (LCSs) and global citation scores (GCSs) were documented in Table 7. LCS reflects the citations within the downloaded dataset, whereas GCS corresponds to the citations recorded in the WoS database. In 2011, Katz et al. [47] found that *P. gingivalis* was abundant in malignant oral epithelium, hinting at a possible link between this bacterium and gingival squamous cell carcinoma. In 2012, Ahn et al. [30] showed that higher serum *P. gingivalis* IgG might be often associated with increased mortality of orodigestive cancer. For healthy people who did not show significant periodontal disease, orodigestive mortality associated with *P. gingivalis* was also found to be too high. Pushalkar et al. [31] depicted that the significant changes in bacterial diversity in oral mucosa of nontumor and tumor sites in OSCC subjects indicate altered bacterial colonization, which may be related to OSCC. In 2013, Michaud et al. [29] showed that individuals with higher antibody levels against *P. gingivalis* ATTC 53978 had twice the risk of pancreatic cancer than those with lower antibody levels, and inhibition of its growth could reduce the risk of pancreatic cancer. In 2014, Schmidt et al. [25] demonstrated that in oral cancer patients, the abundance of *Firmicutes* (especially *Streptococcus*) and *Actinomycetes* (especially *Rothia*) on the affected side was significantly lower than that of the contralateral normal sample of the same patient. Inaba et al. [34] showed that *P. gingivalis* can promote the invasion of OSCC through the induction and activation of proMMP9. In 2015, Binder et al. [32] showed that periodontal pathogens *P. gingivalis* and *F. nucleatum* could promote tumor progression in oral-specific carcinogenesis models. In 2016, Guerrero-Preston et al. [26] showed that compared with the control group, the microbial richness and diversity of HNSCC patients decreased significantly, and the operation also affected the microbial structure of the patients, which was manifested as a decrease in the measured value of α diversity after operation and an increase in the measured value of α diversity in recurrent patients. A comprehensive review [41] outlined that bacteria, including *P. gingivalis*, *F. nucleatum*, and *Streptococcus* spp., may contribute to oral carcinogenesis by suppressing apoptosis, stimulating cellular proliferation, facilitating cellular invasion, eliciting chronic inflammation, and generating carcinogens. In 2017, Peters et al. [13] found that *Tannerella forsythia* could be associated with a higher risk of an elevated risk of esophageal adenocarcinoma (EAC) and the abundance of *P. gingivalis* might be associated with a heightened risk of ESCC. Zhao et al. [48] showed that oral bacterial taxa associated with periodontitis might be significantly enriched in OSCC samples. Al-Hebshi et al. [49] identified a group of inflammatory bacteria characterized by *F. nucleatum* and *Pseudomonas aeruginosa* associated with OSCC. In 2018, Fan et al. [24] showed that oral pathogens *P. gingivalis* and *Aggregatibacter actinomycetemcomitans* were associated with a higher risk of pancreatic cancer, and *Fusobacteria* and its ciliates were associated with a lower risk of pancreatic cancer. Flemer et al. [18] showed that the OM of CRC patients is unique, and the analysis of oral-related bacteria may be valuable for the detection of CRC. Yang et al. [27] showed that the abundance of *Fusobacterium* in OSCC patients increased, while the number of *Streptococcus*, *Haemophilus*, *Porphyromonas*, and *Actinomycetes* decreased with CA progression. Hayes et al. [9] showed that the high abundance of *Corynebacterium* and *Kingella* in the oral cavity is associated with a reduced risk of HNSCC and has a potential impact on CA prevention. Perera et al. [50] found that OSCC tissues have low species richness and diversity and enriched the characteristics of inflammatory bacteria in OSCC tissues, including lipopolysaccharide (LPS) biosynthesis and peptidase. In 2019, a review [42] summarized some specific species closely related to oral cancer, such as *Streptococcus* sp., *Prevotella* sp., *Fusobacterium* sp., and *P*. *gingivalis*. Regarding the role of OM in the oncogenic process, three mechanisms have been postulated, including bacteria-stimulating chronic inflammation, bacteria-activating NF-κB and bacteria-inhibiting apoptosis, and bacteria-producing carcinogens. The other review [28] showed that a significant variety of bacterial species residing in the oral cavity, including *P. gingivalis* and *F. nucleatum*, contribute to chronic inflammation, which ultimately may result in the development of oral cancer. The bacterial products and their metabolic derivatives have the potential to induce lasting genetic alterations in the epithelial cells. In 2020, Zhang et al. [51] compared the tumor site and normal tissue microbial composition in patients with OSCC and found that the distribution of oral bacteria showed significant differences between CA and normal tissues, which may be a diagnostic indicator and therapeutic target.

### 3.6. Keywords Analysis in OM/CA Research

#### 3.6.1. High-Frequency Keywords Analysis

**Figure 7:**
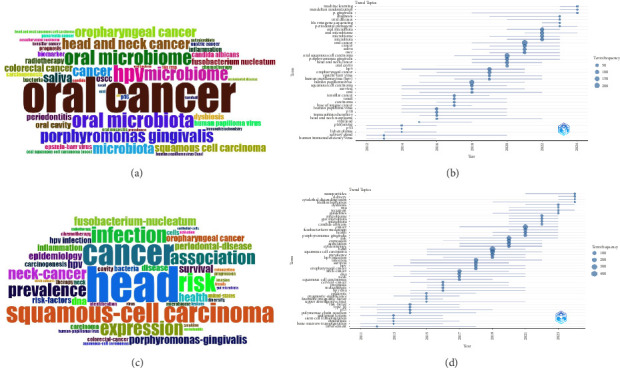
(a) Common Author Keywords in OM/CA research. (b) Evolutionary trends of common Author Keywords over time in OM/CA (circle size represents number of occurrences). (c) Common Keywords Plus in OM/CA. (d) Evolutionary trends of common Keywords Plus over time in OM/CA (circle size indicates number of occurrences).

In this study, we gathered a total of 3185 author's keywords and 2873 keywords plus derived from the OM/CA publications. The highly frequent author's keywords (Figure 7(a)) included “oral cancer,” “oral squamous cell carcinoma,” “head and neck cancer,” “oropharyngeal cancer,” “colorectal cancer,” “tonsillar cancer,” “gastric cancer,” “pancreatic cancer,” “oral microbiota,” “*Human papillomavirus*,” “*Porphyromonas gingivalis*,” “*Fusobacterium nucleatum*,” “*Candida albicans*,” “*Epstein-barr virus*,” “periodontitis,” “radiotherapy,” “chemotherapy,” “oral mucositis,” “biomarker,” “inflammation,” “prognosis,” “carcinogenesis,” “survival,” “smoking,” “alcohol,” etc. The most frequent keywords plus (Figure 7(c)) included “cancer,” “squamous-cell carcinoma,” “prevalence,” “expression,” “association,” “*Fusobacterium-nucleatum*,” “*Porphyromonas-gingivalis*,” “risk,” “infection,” “oropharyngeal cancer,” “periodontal-disease,” “HPV infection,” “survival,” “inflammation,” “carcinogenesis,” “colorectal-cancer,” “chemotherapy,” etc.

#### 3.6.2. Keyword Trend Analysis

According to the keyword trend analysis, future research directions and hotspots can be predicted to support scientific research decision-making. As shown in Figures 7(b) and 7(d), the main author keywords included “mendelian randomization,” “machine learning,” “*P. gingivalis*,” “periodontal pathogens,” “oral diseases,” “oral microbiota,” “diagnosis,” “head and neck cancer,” “oral squamous cell carcinoma,” and “oral cancer.” The main keywords plus included “nanoparticles,” “biofilm formation,” “cytolethal distending toxin,” “guidelines,” “dysbiosis,” “*Candida-albicans*,” “*Porphyromonas gingivalis*,” “*Fusobacterium-nucleatum*,” “gut microbiota,” “epidemiology,” “cancer,” and “association.”

#### 3.6.3. Cluster Analysis of High-Frequency Keywords

**Figure 8:**
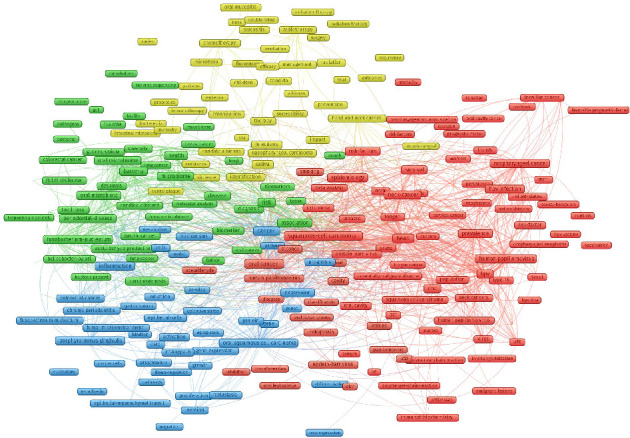
Cluster analysis of keywords within OM/CA research (each cluster of keywords forms a distinct category represented by a unique color. These various colors signify different clusters, and the size of each circle visually indicates the frequency of occurrence of the corresponding keyword).

By grouping similar keywords together, cluster analysis reveals underlying trends, patterns, and emerging themes within the field. This approach facilitates a deeper understanding of the evolution and direction of OM/CA research, enabling researchers to identify key areas of focus, potential gaps in knowledge, and future research opportunities. Based on the co-occurrence of keywords with a frequency of at least 10, a cluster analysis was performed to evaluate the associations and interconnections among these keywords. The outcomes of this cluster analysis are clearly presented in Figure 8.

Cluster 1 (green nodes) was related to specific OM in CA, including *Fusobacterium nucleatum*, *Porphyromonas gingivalis*, *Streptococcus anginosus*, *Helicobacter pylori* (HP), *Candida albicans*, periodontal pathogens, esophageal cancer, GC, lung cancer (LC), and pancreatic cancer.

Cluster 2 (red nodes) focused on the links between OM (HPV and *Epstein–Barr virus* [EBV]) and CA (HNC, oropharyngeal cancer, oral cavity cancer, tongue cancer, cervical cancer, squamous cell carcinoma), and OM can predict tumorigenesis and screening for CA.

Cluster 3 (yellow nodes) focused on the link between OM, anticancer treatment-related adverse reactions (such as chemotherapy and radiotherapy, mucositis, oral mucositis, and bacteremia) and management (prevention, probiotics), and CA prognosis (such as outcomes, recurrence, efficacy, and resistance).

Cluster 4 (blue nodes) focused on the mechanisms by which the OM affects CA, including inflammation (NF-kappa-b, Toll-like receptors), immunity (such as T cells, dendritic cells, epithelial cells, and e-cadherin), apoptosis, gene expression (overexpression, activation, downregulation, and proliferation), epithelial–mesenchymal transition, and oxidative stress.

## 4. Discussions

### 4.1. Overview of Publications in OM/CA

Over the past few decades, as our understanding of OM has deepened and evidence has mounted indicating that OM might serve as a key modulator influencing the occurrence and progression of CA, the OM/CA research has seen a gradual surge. From the annual Np perspective, the years 2010–2017 marked a phase of gradual development, whereas 2018–2020 witnessed a period of rapid advancement, followed by a stage of sustained and steady development from 2021 to 2024. In 2010, a tool QIOME [6] unlocked new direction for OM study. An increasing number of countries embarked on developing microbiome projects, thereby fostering advancements in OM/CA research. In 2018, two pivotal studies [18, 24] in Gut highlighted the significance of OM as a promising biomarker and established precise disease-predictive models. Consequently, OM/CA research has garnered growing attention from scholars since then. High-volume journals are mainly concentrated on journals related to oral cavities, CA, and microbiology. The significance of journals with high publication volumes lies in their capacity to disseminate a vast amount of research findings promptly. They serve as essential platforms for academics to share their work widely and foster interdisciplinary communication. The drawbacks of journals with high publication volumes include potentially lower academic rigor due to the increased volume of submissions.

Most of these publications originated from the United States and China. Both the United States and China have substantial government and private funding dedicated to research in various fields, including oncology, microbiology, and molecular sciences. These fundings may support not only the conduct of research but also its publication in high-impact journals. Therefore, the main research institutions also came from these countries. Furthermore, notably, research output and funding addressing the OM–CA nexus remain disproportionately concentrated in high-income countries (HICs). Meanwhile, lower-middle-income (LMICs) and low-income countries (LICs)—which bear the highest burden of microbiome-associated CAs like oral carcinoma—contribute minimally due to economic constraints and resource limitations. This geographic disparity perpetuates inequities in evidence generation and impedes translational applications of microbiome science for global CA prevention and control.

Different authors have different research directions in OM/CA research. For example, Dalianis Tina, Nasman Anders, and Ramqvist Torbjorn from *Karolinska University Hospital* focused on the effect of HPV on OSCC, tongue squamous cell carcinoma, tonsillar cancer and base of tongue cancer had the most TC and published many high-cited papers [37, 52]. Pientong Chamsai and Ekalaksananan Tipaya from Khon Kaen University focused on the effect of EBV and HPV on OSCC [53, 54]. Pan Yaping from China Medical University focused on the effect of *P. gingivalis* and *F. nucleatum* on OSCC [55, 56]. By tracking their latest research results, we may be able to keep abreast of the latest developments in OM/CA fields. The significance of highly cited papers lies in their recognized contribution to advancing knowledge in a particular field. They serve as key references that guide further research and innovation. Historical cited papers in OM/CA research provided a foundation for understanding the historical development and evolution of a field.

### 4.2. Current Research Status and Hotspots

The clustering analysis of keywords and highly cited papers revealed the current status and hotspots in OM/CA research, including: (1) elucidating the impact of OM or specific OM on the initiation and progression of CA; (2) investigating alterations in OM characteristics of CA; (3) assessing the utility of OM in CA screening and outcomes; (4) examining the influence of OM on CA treatment outcomes and related adverse reactions; and (5) exploring the mechanisms of OM involved in CA.

#### 4.2.1. The Effect of Specific OM on Tumorigenesis and Progression

The composition of OM, encompassing bacteria, fungi, and viruses, could facilitate carcinogenesis and tumor progression. Specific OM such as *P*. *gingivalis*, *F*. *nucleatum*, *Candida albicans*, *HPV*, and *EBV* may be linked to the initiation and advancement of tumors. Notably, periodontal pathogens *P*. *gingivalis* and *F*. *nucleatum* have been hot topics of interest in recent years.

1. *Porphyromonas gingivalis* (*P. gingivalis*): *P. gingivalis* is a significant periodontal pathogen that not only causes periodontitis but may also be associated with various systemic diseases. *P. gingivalis* has been implicated in facilitating the invasion and progression of OSCC [34], and it may serve as a biomarker for microbe-associated mortality risk in orodigestive cancers [30]. *P. gingivalis* was abundant in malignant oral epithelium, hinting at a potential association between this bacterium and gingival squamous cell carcinoma [47]. Furthermore, *P. gingivalis* might be associated with the progression and overall survival of ESCC and could potentially be used as a biomarker for esophageal cancer [33, 57]. *P. gingivalis* not only could promote the proliferation and migration of ESCC cells [58] but also might predict local recurrence following endoscopic submucosal dissection in early ESCC [59]. Additionally, *P. gingivalis* could exacerbate esophageal cancer while promoting resistance to neoadjuvant chemotherapy [60].
2. *Fusobacterium nucleatum* (*F. nucleatum*): *F. nucleatum* is a prevalent oral opportunistic bacterium, enriched in periodontal diseases, oral cancer, and systemic diseases [61]. This bacterium exerts its oncogenic potential by inducing DNA damage and stimulating cell proliferation in oral cancer cells [56]. Furthermore, *F. nucleatum* could directly interact with oral epithelial cells via Toll-like receptors, thereby stimulating tumorigenesis [32]. Salivary levels of *F. nucleatum* might emerge as a potential diagnostic biomarker for both GC [62] and CRC [63], and CRC-associated *F. nucleatum* may derive from the oral cavity [64]. Moreover, *F. nucleatum* could promote cisplatin resistance and migration of OSCC by upregulating Wnt5a-mediated NFATc3 expression [65]. *F. nucleatum* can foster CA development through enhanced cell proliferation, increased cellular invasion, induction of chronic inflammation, and immune evasion mechanisms [41].
3. *Candida albicans* (*C. albicans*): *C. albicans* could play a role in carcinogenic events within the oral cavity [66]. A dysbiotic mycobiome dominated by *C. albicans* had been identified within OSCC tissues [67]. An elevated burden of *C. albicans* could foster an oncogenic phenotype and accelerate the progression of OSCC [68]. Moreover, *C. albicans* isolates obtained from potentially carcinogenic oral diseases might have the capacity to produce mutagenic levels of acetaldehyde [69].
4. HPV: A potentially causal relationship could exist between HPV and OSCC and OPMD [45]. High incidences of HPV infection have been detected in OSCC, indicating that HPV could elevate the risk of OSCC tumorigenesis [54, 70]. Salivary high-risk HPV DNA serves as a potential biomarker for HPV-driven HNC [71]. Furthermore, high-risk HPV infection, particularly HPV-16, might be a contributing factor to the development of OSCC [72].
5. EBV: Rather than initiating tumors, EBV likely acts as a promoter of tumor progression, contributing to the malignant phenotypes observed in EBV-positive CAs [73]. A meta-analysis indicates a statistical association between EBV infection and an increased risk of OSCC [74]. Furthermore, the prevalence of EBV in OSCC cases appears to be heightened by betel quid chewing, suggesting that both factors may act synergistically as significant etiological risk factors for OSCC [53].

#### 4.2.2. Changes of OM Characteristics in CA

##### 4.2.2.1. OM and Head and Neck Cell Cancer

OM features are linked to HNC. Hayes et al. [9] observed a link between higher oral abundance of commensal *Corynebacterium* and *Kingella* and a lower HNSCC risk. Wu et al. [75] reported that increased taxonomic alpha-diversity, oral fungi presence, and the abundance of various microbial species, including periodontal pathogens in the red and orange complex, were associated with decreased HNC risk. Moreover, there might be a potential association between OM and OSCC [76]. OSCC patients exhibited significantly increased abundances of *F. nucleatum*, *Capnocytophaga sputigena*, *Porphyromonas endodontalis*, and *Gemella haemolysans* compared to controls [77]. Bacterial richness and diversity were notably higher in tumor sites than in normal tissue of OSCC patients, with CA tissues enriched in *F. nucleatum*, *Prevotella intermedia*, and *Aggregatibacter segnis* [51]. OSCC tissues tended to show lower species richness and diversity, with enrichment of *Campylobacter concisus*, *Prevotella salivae*, *Prevotella loescheii*, and *Fusobacterium* [50]. The frequency of isolation of total aerobes, total anaerobes, coliforms, and gram-negative anaerobic bacteria was significantly higher in OSCC patients than in HCs [78]. In addition, HPV has been proposed as a risk factor in the development of OSCC, and other oncogenic virus species such as EBV and HSV Type 1 may also be involved in oral carcinogenesis [79].

##### 4.2.2.2. OM and CAs of Other Body Sites

Research suggests a potential link between the composition of OM and the risk of certain CAs, including (1) esophagus cancer (EC): Variations in OM abundance distinguish EC patients from HCs. EC patients show increased *Firmicutes*, *Negativicutes*, *Selenomonadales*, *Prevotellaceae*, and decreased *Proteobacteria*, *Betaproteobacteria*, and *Neisseriales* [80]. Furthermore, *P. gingivalis* and *Tannerella forsythia* may be linked to a higher risk of EAC, while *Neisseria* and *Streptococcus pneumoniae* depletion might be correlated with lower EAC risk [13]. (2) GC: Tongue-coating microbiome may aid early GC detection. *Streptococcus* could trend with a higher risk of GC, and *Neisseria*, *Prevotella*, and *Porphyromonas* showed decreased risk [14]. OM including *Haemophilus*, *Neisseria*, *Parvimonas*, *Peptostreptococcus*, and *Neisseria* significantly decreased in GC patients [81]. OM–HP interactions may provide GC screening insights. Integration of OM and HP might manifest as a potential method for assessing GC risk [82]. Furthermore, a study in *Cell* discovered that *Streptococcus anginosus* can promote GC by directly interacting with the TMPC–ANXA2–MAPK axis of gastric epithelial cells [83]. (3) Pancreatic carcinoma (PC): Pathogenic oral bacteria are found in pancreatic tumors [84]. *Haemophilus*, *Porphyromonas*, *Leptotrichia*, and *Fusobacterium* can differentiate patients with pancreatic head cancer (PHC) from HCs, while *Streptococcus* and *SR1* can distinguish PHC patients from those with liver cancer [15]. *P. gingivalis* and *Aggregatibacter actinomycetemcomitans* could increase PC risk, while *Fusobacteria* and *Leptotrichia* could decrease it [24]. Saliva microbiome was able to distinguish PC patients from HCs [85] and represents a valuable tool for PC detection [23]. (4) CRC: OM from periodontal disease can alter GM, potentially contributing to CRC [86]. Similar bacterial networks at both oral and colonic mucosal surfaces, with a high abundance of *Lachnospiraceae* negatively correlating with colonic colonization by OM, hint at its protective role against CRC [18]. Moreover, the saliva microbiome of HCs exhibited a distinct α diversity and clustered β diversity compared to CRC patients. *Bifidobacterium* might be a potential CRC biomarker, while *Fusobacterium*, *Dialister*, *Catonella*, etc., might distinguish CRC patients [87]. (5) LC: Some studies have hinted at a possible association between alterations in oral bacterial populations and the incidence of LC. LC patients might show a reduced microbial diversity and richness compared to HCs [88]. *Streptococcus* abundance might be associated with the risk of LC [89]. Moreover, non-small-cell LC patients show an increased *Veillonella* and *Streptococcus*, with decreased *Fusobacterium*, *Prevotella*, etc. [90]. Furthermore, OM may link periodontitis to malignancies [91].

#### 4.2.3. The Role of OM in Screening and Prognosis of CA

The OM holds significant potential for integration into clinical diagnostic procedures and screening programs of CA. OM can be used as a key biomarker for noninvasive detection and diagnosis of CA such as OSCC [12], nasopharyngeal carcinoma [92], oral and oropharyngeal cancers [93], esophageal cancer [13], GC [14], gastrointestinal cancer [94], pancreatic cancer [15], and CRC [16].

Eun et al. [95] showed that the random forest (RF) model based on OM features showed a modestly high accuracy for predicting metastasis in OSCC patients, with an area under the curve (AUC) of 0.89. Yang et al. [27] found that a bacterial-marker panel demonstrated an AUC value of 0.956 in distinguishing patients with Stage 4 OSCC patients from the HCs. Lim et al. [93] found that a biomarker panel of OM demonstrated a remarkable ability to predict the occurrence of oral and oropharyngeal cancers, achieving an AUC of 0.98, as well as a sensitivity of 100% and a specificity of 90%.

Wei et al. [96] reported that the AUC values under the receiver operating characteristic (ROC) curves for predicting ESCC patients using *Streptococcus salivarius*, *F. nucleatum*, *P. gingivalis*, and their combination were 69%, 56.5%, 61.8%, and 76.4%, respectively. Huang et al. [81] found that GC could be distinguished from patients with superficial gastritis (SG) and atrophic gastritis (AG) by the RF model based on the salivary microbiota (AUC = 0.91). Xu et al. [97] revealed that the differential OM between GC patients and HCs. By RF analysis, the combination of six bacterial genera was the optimal predictive model to distinguish GC patients from HCs, with an AUC value of 0.85. Zhang et al. [16] showed that OM composition and diversity were significantly different among the CRC, colorectal adenoma, and HCs. A RF model was developed to differentiate CRC patients from HCs, demonstrating robust classification potential with an AUC of 76.42% in the discovery cohort and 63.86% in the validation cohort.

Furthermore, evidence suggests that OM may also be associated with CA prognostic outcomes. There was a significant difference in beta-diversity between the metastasis and no metastasis groups in OSCC patients, and the metastasis group was enriched in the *Prevotella*, *Stomatobaculum*, *Bifidobacterium*, *Peptostreptococcaceae*, *Shuttleworthia*, and *Finegoldia*. The RF model showed a modestly high accuracy for predicting metastasis [95]. *Fusobacterium periodonticum*, *Parvimonas micra*, *Streptococcus constellatus*, *Haemophilus influenza*, and *Filifactor alocis* were associated with OSCC, and they progressively increased in abundance from Stage 1 to Stage 4 [27]. Additionally, a meta-analysis reveals that the composition of OM could potentially serve as a key indicator for predicting survival outcomes such as overall survival and disease-specific survival in individuals with CA [17].

#### 4.2.4. The Effect of OM on Treatment of CA

##### 4.2.4.1. Efficacy of CA Treatment

OM could modulate the treatment response in CA patients. Illustratively, *F. nucleatum* has been shown to augment the chemoresistance of CA cells [98], while *P. gingivalis* infection has been implicated in fostering resistance to neoadjuvant chemotherapy [60]. Furthermore, OM might hold potential significance in dictating the sensitivity and therapeutic responsiveness of OSCC patients to chemotherapy [99]. OM could influence the effectiveness and prognosis of radiotherapy in CRC [100].

##### 4.2.4.2. Adverse Reactions of CA Treatment

Oral mucositis, a prevalent, agonizing, and debilitating adverse effect of CA therapy, leads to ulcer formation and microbial colonization, thereby impairing quality of life and treatment adherence. Variations in OM during the treatment period were linked to the emergence of oral mucositis [101].

1. Radiotherapy-induced oral mucositis: OM imbalance may exacerbate oral mucositis severity among patients undergoing radiotherapy [102]. During radiotherapy, the bacterial community undergoes shifts, particularly with an increase in Gram-negative bacteria (GNB), which affect patient susceptibility to OM disturbances [103]. Furthermore, there is a potential link between OM and delayed healing of oral mucositis. *Actinobacteria* and *Veillonellaceae* may serve as biomarkers for predicting this risk [104].
2. Chemotherapy-related oral mucositis: Patients who developed oral mucositis may have greater microbial diversity at diagnosis and underwent more substantial bacterial alterations due to chemotherapy before mucositis occurred [105]. The shifts in the oral mucositis-associated bacteriome involve a depletion of common health-promoting commensals and an enrichment of GNB such as *F. nucleatum* and *Prevotella oris* [106]. In addition, *C. albicans* and HSV-1 also act in chemotherapy-induced oral mucositis [107].

#### 4.2.5. Carcinogenic Mechanisms of OM

The underlying mechanisms of OM-related carcinogenesis involve a complex process, including the colonization and persistence of pathogenic or oncogenic bacteria, disruption of the epithelial barrier, bacteria translocation, the presence of carcinogenic bacterial metabolites and toxins, the induction of chronic inflammation and immunosuppression, triggering DNA damage, cell proliferation, and immune evasion to promote oral and gastrointestinal cancers [5, 10, 41, 42].

##### 4.2.5.1. Induction of Inflammation

Inflammation can drive all stages of CA by modifying the composition of the OM and stimulating the growth of genotoxic species. Chronic inflammation is acknowledged as a potential pathway by which bacteria may contribute to the development of oral cancer, as evidenced by the strong association between periodontitis and an increased risk of OSCC. *P. gingivalis* and *F. nucleatum* could contribute to chronic inflammation, which may result in the development of oral cancer [28]. *Streptococcus mutans* might foster tumor metastasis by inducing vascular inflammation [108]. Oncobacteria facilitate the progression of OSCC through heightened expression of inflammatory cytokines, including IL-6, IL-8, tumor necrosis factor-alpha (TNF-α), and interferon-gamma (IFN-γ) [109]. *P. gingivalis* could enhance IL-6 production, which in turn might promote epithelial–mesenchymal transition and the recruitment of myeloid-derived suppressor cells [57]. Furthermore, *P. gingivalis* could promote colorectal tumorigenesis by fostering a proinflammatory tumor microenvironment via the activation of the NLRP3 inflammasome [110].

##### 4.2.5.2. Production of Carcinogens

Substances produced by OM might also be associated with carcinogenesis. OM can impact CA through carcinogenic metabolites, such as reactive oxygen species (ROS), volatile sulfur compounds (VSCs), LPS, and lactic acid [42, 111]. ROS are microbiota-induced metabolic byproducts and involved in CA progression [111]. Certain OM can produce VSCs, which exhibit toxicity to tissues even in minute concentrations. LPS, a pathogenic substance commonly found in many anaerobic oral bacteria, can trigger inflammatory processes that are intricately linked to inflammation-associated carcinogenesis. *P. gingivalis*–derived LPS could promote the proliferation and migration of glioma cells and modulate the proliferation and invasion of HNC cell lines [112]. Certain OM, such as *Lactobacillus*, *Lactococcus*, *Bifidobacterium*, and *Streptococcus*, produce lactic acid, which may contribute to the acidic and hypoxic tumor microenvironment, enhancing metastatic efficiency [42].

##### 4.2.5.3. Pathogen‐Induced Immunosuppression

The microbiota modulates innate immune signaling and protective immunity against CA [113], with microbiota-associated immunosuppression being a crucial factor in carcinogenesis. Periodontitis-derived OM can facilitate OSCC progression through gamma delta T-cell activation [114]. HPV plays a pivotal role in HNC, which correlates with tumor-infiltrating lymphocytes [115]. CD8+ and CD4+ tumors–infiltrating lymphocytes are associated with HPV status and clinical outcomes in tonsillar and base of tongue squamous cell carcinoma [52]. Moreover, *F. nucleatum* could promote a proinflammatory tumor microenvironment by recruiting tumor-infiltrating immune cells. Additionally, *F. nucleatum* could inhibit natural killer cells and various T lymphocytes by binding Fap2 to T-cell immunoreceptors, thereby impairing antitumor immune activities [116]. Similarly, *P. gingivalis* can suppress the host immune system and promote tumor progression by creating a tumor-promoting immune microenvironment [117].

In a nutshell, chronic inflammation from oral pathogens (e.g., *F. nucleatum*, *P. gingivalis*) activates proinflammatory pathways (NF-κB, NLRP3), releasing cytokines that induce DNA damage. Their carcinogenic metabolites (H2S, formate) disrupt epithelial barriers, while immunosuppression in the tumor microenvironment enables pathogen survival and immune evasion. Notably, no single or consortium of oral bacteria directly initiates carcinogenesis; their role lies in metabolic/immune modulation. Current research lacks standardized gnotobiotic models, and demonstrated mechanisms often reflect adaptive survival in tumors rather than direct malignancy induction. Carcinogenesis arises from synergistic microbial–host interactions [41, 42].

### 4.3. Emerging Research Trends in OM/CA Research

Through keyword trend analysis, we found that researchers are uncovering new insights into the role of OM in CA onset, development, diagnosis, and treatment. These efforts hold the potential to revolutionize CA prevention, diagnosis, and treatment strategies in the future.

#### 4.3.1. Mendelian Randomization (MR)

MR studies leverage genetic variants as instrumental variables to investigate causal relationships between exposures and outcomes, which plays a potential role in understanding the causal relationship between OM and CA, and can provide valuable insights into the causal mechanisms linking the OM to CA risk [118–120]. By using genetic variants as instrumental variables, MR can help isolate the effects of some factors, minimizing the influence of confounding variables. Thus, MR application in OM/CA research can inform and strengthen the scientific basis for developing future screening strategies that consider microbial influences.

#### 4.3.2. ML

The role of clinical and translational applications of OM in CA screening, particularly when enhanced by ML, is increasingly recognized as crucial. By analyzing the complex interplay of microbial species within the oral cavity, ML algorithms can identify distinct patterns and signatures that may be indicative of underlying cancerous conditions [121]. This advanced computational approach allows for more precise and efficient screening, enabling early detection and potentially improving treatment outcomes. The integration of ML with OM research opens new possibilities for developing noninvasive, cost-effective, and highly accurate CA screening tools [122, 123].

#### 4.3.3. Biofilm Formation

Biofilm formation within OM, a complex community of microorganisms residing in the mouth, has emerged as an intriguing area of study in relation to CA. These biofilms, composed of bacteria, fungi, and other microorganisms embedded in a matrix of extracellular polymeric substances, can influence local inflammation and immune responses. Recent research suggests that alterations in OM and its biofilm-forming capabilities may contribute to the development and progression of certain types of CA, particularly those involving the head and neck region [124, 125]. Understanding the intricate relationship between biofilm formation, OM, and CA could lead to novel therapeutic approaches aimed at modulating this microbial environment to prevent or manage CA.

#### 4.3.4. Cytolethal Distending Toxin (CDT)

CDT, produced by certain OM, has garnered attention in CA research. CDT is known for its ability to induce cell cycle arrest and apoptosis in target cells, making it a potentially significant player in the complex interplay between OM and CA development [126]. Although the exact mechanisms are still being elucidated, it is hypothesized that CDT may contribute to genomic instability and chronic inflammation, both of which are hallmarks of CA [127]. Thus, the presence and activity of CDT-producing bacteria in OM could represent a risk factor for the initiation or progression of certain CAs, necessitating further investigation into this fascinating connection.

#### 4.3.5. Nanoparticles

By serving as carriers for targeted therapies, nanoparticles can facilitate the delivery of CA-fighting agents directly to the site of action, guided in part by the unique microbial landscape of the oral cavity. A study presents bacterial outer membrane vesicle-encapsulated TiO_2_ nanoparticles as a dual-action therapeutic platform for enhanced and targeted treatment of OSCC, potentially improving therapeutic outcomes and reducing adverse effects [128]. Furthermore, they enable the modulation of OM, potentially enhancing antitumor properties or mitigating proinflammatory effects [129]. Nanoparticles hold promise for developing innovative and more effective CA treatment strategies.

### 4.4. Key Research Gaps and Prospects for Future Exploration

Research on the relationship between OM and CA encounters multiple challenges, primarily due to the complexity of the oral microbial ecosystem. Currently, clinicians have very limited recommended uses for employing OM as biomarkers for CA prediction. Most proposed applications remain under investigation, with their potential being explored and considered. There are notable deficiencies in this field. First, the complex mechanisms linking OM to CA prognosis are not yet fully understood. Although studies have suggested associations between OM imbalances and the onset, progression, and prognosis of certain types of CA, most of these findings are still at a preliminary stage and require further validation. Establishing a direct causal link between OM and CA remains challenging. Second, the reproducibility and generalizability of these findings across different populations and geographical regions remain uncertain. Research conducted in one setting may not necessarily translate seamlessly to others, due to variations in OM compositions and CA incidence rates among diverse populations. Furthermore, the technical challenges associated with accurately measuring and interpreting OM data also pose significant hurdles. The sensitivity and specificity of current diagnostic tools for identifying specific OM biomarkers need improvement to ensure reliable and consistent results. Lastly, the ethical and regulatory considerations surrounding the use of OM as biomarkers are still being navigated. Ensuring patient privacy, obtaining informed consent, and adhering to relevant guidelines and regulations are crucial steps that must be taken seriously.

In addition, personalized medicine approaches consider individual microbial variations, enhancing intervention effectiveness. The therapeutic modulation of OM in CA management holds immense significance, as it not only forestalls the onset and progression of CA but also bolsters clinical efficacy and mitigates adverse events. Currently, the primary strategies for intervening in OM to address CA encompass probiotics, diet, lifestyle adjustments such as quitting smoking and limiting drinking, and oral microbiota transplantation (OMT). Probiotics can augment the antitumor efficacy of chemotherapy [130] and have been linked to favorable clinical outcomes in the context of immunotherapy [131]. Utilizing OMT and animal models that emulate human oral dysbiosis can facilitate the reconstruction of the OM in mice, enabling the observation of changes in disease phenotypes [132]. OMT has also shown promise in mitigating radiotherapy-induced oral mucositis [133]. In addition, public health interventions, such as promoting oral hygiene and dental checkups, are crucial for maintaining a healthy OM and reducing CA risk. Despite these challenges, ongoing efforts are making significant strides in understanding and potentially preventing CA related to OM, paving the way for more tailored and effective preventive strategies.

### 4.5. Limitations of the Research

Our study still has some potential drawbacks. First, the study exclusively searched and included papers from the WoSCC, which may not encompass all studies published across multiple databases globally. This may lead to incompleteness in the results. Second, bibliometrics primarily relies on quantitative data, such as citation counts and publication frequencies, which may not fully capture the quality or impact of research. This can lead to a bias toward quantity over quality, potentially rewarding researchers who produce a high volume of work rather than those who produce fewer but more influential studies. By focusing on high-cited papers and high-IF papers, the study aimed to capture key trends and insights that may have been missed by the bibliometric tools. Third, bibliometrics is limited by the availability and completeness of data, and there may be delays in the indexing and updating of these databases. This can result in incomplete or outdated information and limit its accuracy.

## 5. Conclusion

In the past 15 years, the OM/CA research had increased rapidly, and China and the United States had made important contributions to it. Karolinska Institutet and Harvard University had the maximum Np. Tina Dalianis and Anders Naesman emerged as the most prolific authors, while Oral Oncology garnered the highest Np, and Gut received the greatest total number of citations. OM is intricately linked to oral and gastrointestinal cancer and holds potential as a noninvasive biomarker for the identification and early screening of CA patients, predicting CA prognosis, and assessing treatment efficacy. In the oral flora, *F. nucleatum*, *P. gingivalis*, *Candida albicans*, HPV, and EBV have been implicated in close association with the initiation and progression of tumors. Notably, the most studied two oral bacteria *P. gingivalis* and *F. nucleatum* do not initiate carcinogenesis, but chronic periodontitis may facilitate carcinogenesis due to immunosuppression and overburdened immune system. On the one hand, it needs to eliminate neoplastic cells. On the other hand, it needs to eliminate the putative periodontal pathogens. Association of putative periodontal pathogens in oral, CRC, and pancreatic cancers needs revisiting in this scenario. Modulating the OM not only presents an opportunity for CA prevention but also could enhance the clinical outcomes of CA treatment. Thus, gaining a comprehensive understanding of the mechanisms underlying the interaction between OM and CA, followed by remodeling the OM for CA treatment, emerges as a fascinating and promising research direction. Despite the promising potential of OM as biomarkers for CA prediction, there are still significant deficiencies and challenges that need to be addressed before they can be widely adopted in clinical practice. In summary, this study illuminates the global research landscape of OM/CA research, providing scholars with a deeper understanding of its developmental trends and offering a comprehensive perspective for further in-depth exploration.

## Figures and Tables

**Table 1:** The top 10 most productive journals in OM/CA.

Rank	Journals	Np	Citations	H-index	2023 IF	2023 JCR
1	Oral Oncology	47	1876	25	4	Q1
2	Frontiers in Cellular and Infection Microbiology	45	1292	18	4.6	Q1
3	Cancers	39	640	15	4.5	Q1
4	International Journal of Molecular Sciences	32	533	11	4.9	Q1
5	Frontiers in Microbiology	25	875	15	4	Q2
6	Journal of Oral Pathology & Medicine	25	554	12	2.7	Q1
7	Scientific Reports	24	1136	14	3.8	Q1
8	Journal of Oral Microbiology	22	717	11	3.7	Q2
9	Frontiers in Oncology	21	295	9	3.5	Q2
10	PLoS One	21	1014	14	2.9	Q1

**Table 2:** The top 10 most productive authors in OM/CA.

Rank	Authors	Np	Citations	H-index	Affiliations	Countries
1	Dalianis, Tina	26	1199	18	Karolinska University Hospital	Sweden
2	Naesman, Anders	25	1108	17	Karolinska Institutet	Sweden
3	Ramqvist, Torbjorn	22	1146	17	Karolinska University Hospital	Sweden
4	Haeggblom, Linnea	14	443	10	Karolinska University Hospital	Sweden
5	Gao, Shegan	14	528	8	Henan University of Science & Technology	China
7	Pientong, Chamsai	13	162	8	Khon Kaen University	Thailand
8	Ekalaksananan, Tipaya	13	162	8	Khon Kaen University	Thailand
6	Munck-Wikland, Eva	12	908	12	Karolinska Institutet	Sweden
9	Pan, Yaping	12	408	9	China Medical University	China
10	Hammarstedt-Nordenvall, Lalle	11	677	10	Karolinska Institutet	Sweden

**Table 3:** The top 10 most productive institutions in OM/CA.

Rank	Institutions	Np	Citations	H-index
1	Karolinska Institutet (Sweden)	54	2119	22
2	Harvard University (USA)	49	2875	25
3	University of California System (USA)	37	1714	13
4	University of Texas System (USA)	35	1309	16
5	Karolinska University Hospital (Sweden)	34	1452	19
6	Sichuan University (China)	34	815	15
7	National Institutes of Health (USA)	30	2075	17
8	State University System of Florida (USA)	29	1163	16
9	Sun Yat Sen University (China)	28	577	13
10	UTMD Anderson Cancer Center (USA)	28	979	14

**Table 4:** The top 10 most productive countries in OM/CA.

Rank	Countries	Np	Citations	Average citation	H-index
1	USA	402	15,113	37.59	67
2	China	366	8687	23.73	48
3	India	156	2197	14.08	26
4	Japan	125	2076	16.61	24
5	Italy	91	2155	23.68	25
6	Brazil	74	1150	15.54	19
7	Sweden	74	2687	36.31	24
8	Australia	63	2088	33.14	27
9	UK	57	2082	36.53	23
10	Germany	57	1403	24.61	19

**Table 5:** The top 20 highly cited original research in OM/CA.

No.	DOI	Cancer types	Methods	First author	Year	Journals	IF	JCR	Citations
1	10.1136/gutjnl-2016-312580	Pancreatic cancer	16S rRNA	Fan, Xiaozhou	2018	Gut	23	Q1	516
2	10.1136/gutjnl-2011-300784	Pancreatic cancer	qPCR	Farrell, James J.	2012	Gut	23	Q1	465
3	10.1136/gutjnl-2017-314814	CRC	16S rRNA	Flemer, Burkhardt	2018	Gut	23	Q1	404
4	10.1136/gutjnl-2012-303006	Pancreatic cancer	Antibody detection	Michaud, Dominique S.	2013	Gut	23	Q1	299
5	10.18632/oncotarget.4209	OSCC	—	Gallimidi, Adi Binder	2015	Oncotarget	—	—	286
6	10.1158/0008-5472.CAN-17-1296	Esophageal cancer	16S rRNA	Peters, Brandilyn A.	2017	Cancer Res.	12.5	Q1	267
7	10.1016/j.oraloncology.2012.07.002	OSCC	qPCR	Lingen, Mark W.	2013	Oral Oncol.	4	Q1	260
8	10.1371/journal.pone.0098741	Oral cancer	16S rRNA	Schmidt, Brian L.	2014	PLoS One	2.9	Q1	258
9	10.1038/s41598-017-11779-9	OSCC	16S rRNA	Zhao, Hongsen	2017	Sci Rep	3.8	Q1	248
10	10.1093/carcin/bgs112	Orodigestive cancer	Antibody detection	Ahn, Jiyoung	2012	Carcinogenesis	3.3	Q2	244
11	10.3389/fcimb.2019.00476	Oral cancer	16S rDNA	Zhang, Ling	2020	Front. Cell. Infect. Microbiol.	4.6	Q2	233
12	10.3389/fmicb.2018.00862	OSCC	16S rRNA	Yang, Chia-Yu	2018	Front. Microbiol.	4	Q2	225
13	10.1021/acs.nanolett.5b00570	—	—	Hu, Qinglian	2015	Nano Lett.	9.6	Q1	225
14	10.1186/1471-2180-12-144	OSCC	16S rRNA	Pushalkar, Smruti	2012	BMC Microbiol.	4	Q2	225
15	10.18632/oncotarget.9710	Oral cancer	16S rRNA	Guerrero-Preston, Rafael	2016	Oncotarget	—	—	223
16	10.1186/s13027-016-0049-x	ESCC	16S rDNA	Gao, Shegan	2016	Infect. Agents Cancer	3.1	Q3	221
17	10.1001/jamaoncol.2017.4777	HNSCC	16S rRNA	Hayes, Richard B.	2018	JAMA Oncol.	22.5	Q1	215
18	10.1021/acs.nanolett.7b05323	—	—	Fan, Jin-Xuan	2018	Nano Lett.	9.6	Q1	206
19	10.1002/ijc.24994	Tongue cancer	—	Attner, Per	2010	Int. J. Cancer	5.7	Q1	182
20	10.1038/s41598-017-02079-3	OSCC	16S rRNA	Al-hebshi, Nezar Noor	2017	Sci Rep	3.8	Q1	177

**Table 6:** The top 10 highly cited reviews in OM/CA.

No.	DOI	Type	First author	Year	Journals	IF	JCR	Citations
1	10.1177/1533033819867354	Narrative review	Chattopadhyay, Indranil	2019	Technol. Cancer Res. Treat.	2.7	Q3	242
2	10.1111/j.1601-0825.2011.01792.x	Systematic review	Syrjanen, S.	2011	Oral Dis.	2.9	Q1	235
3	10.3390/microorganisms7010020	Narrative review	Karpinski, Tomasz M.	2019	Microorganisms	4.1	Q2	231
4	10.1371/journal.ppat.1003933	Narrative review	Whitmore, Sarah E.	2014	PLoS Pathog.	5.5	Q1	225
5	10.3389/fimmu.2020.591088	Narrative review	Irfan, Muhammad	2020	Front. Immunol.	5.7	Q2	173
6	10.1007/s10552-011-9892-7	Narrative review	Ahn, Jiyoung	2012	Cancer Causes Control	2.2	Q3	172
7	10.1016/j.biopha.2016.09.082	Narrative review	Gholizadeh, Pourya	2016	Biomed. Pharmacother.	6.9	Q1	167
8	10.3390/ijms20174146	Narrative review	Koliarakis, Ioannis	2019	Int. J. Mol. Sci.	4.9	Q1	164
9	10.3402/jom.v8.32762	Narrative review	Perera, Manosha	2016	J. Oral Microbiology	3.7	Q2	149
10	10.3390/cancers13092124	Narrative review	Park, Se-Young	2021	Cancers	4.5	Q1	129

**Table 7:** The historical direct citation network papers in OM/CA.

No.	DOI	Type	Journals	First author	Year	LCS	GCS
1	10.4248/IJOS11075	Article	Int. J. Oral Sci.	Katz, Joseph	2011	70	150
2	10.1093/carcin/bgs112	Article	Carcinogenesis	Ahn, Jiyoung	2012	70	244
3	10.1186/1471-2180-12-144	Article	BMC Microbiol.	Pushalkar, Smruti	2012	98	225
4	10.1136/gutjnl-2012-303006	Article	Gut	Michaud, Dominique S.	2013	66	299
5	10.1371/journal.pone.0098741	Article	PLoS One	Schmidt, Brian L.	2014	114	258
6	10.1111/cmi.12211	Article	Cell Microbiol.	Inaba, Hiroaki	2014	78	169
7	10.18632/oncotarget.4209	Article	Oncotarget	Gallimidi, Adi Binder	2015	114	286
8	10.18632/oncotarget.9710	Article	Oncotarget	Guerrero-Preston, Rafael	2016	83	223
9	10.3402/jom.v8.32762	Review	J. Oral Microbiology	Perera, Manosha	2016	74	149
10	10.1158/0008-5472.CAN-17-1296	Article	Cancer Res.	Peters, Brandilyn A.	2017	75	267
11	10.1038/s41598-017-11779-9	Article	Sci Rep	Zhao, Hongsen	2017	101	248
12	10.1038/s41598-017-02079-3	Article	Sci Rep	Al-hebshi, Nezar Noor	2017	89	177
13	10.1136/gutjnl-2016-312580	Article	Gut	Fan, Xiaozhou	2018	95	516
14	10.1136/gutjnl-2017-314814	Article	Gut	Flemer, Burkhardt	2018	78	404
15	10.3389/fmicb.2018.00862	Article	Front. Microbiol.	Yang, Chia-Yu	2018	110	225
16	10.1001/jamaoncol.2017.4777	Article	JAMA Oncol.	Hayes, Richard B.	2018	78	214
17	10.1177/0022034518767118	Article	J. Dent. Res.	Perera, M.	2018	72	127
18	10.1177/1533033819867354	Review	Technol. Cancer Res. Treat.	Chattopadhyay, Indranil	2019	75	242
19	10.3390/microorganisms7010020	Review	Microorganisms	Karpinski, Tomasz M.	2019	82	230
20	10.3389/fcimb.2019.00476	Article	Front. Cell. Infect. Microbiol.	Zhang, Ling	2020	96	232

## Data Availability

The original contributions presented in the study are encapsulated within the article and Supporting Information. For any additional queries or inquiries, please reach out to the corresponding authors.

## Supporting Information

A detailed account of the search strategy, including the specific search terms, databases queried, the refinement procedure, and search link, was provided in the Supporting Information.
