# Supplementary material for: Crosstalk Between Oral Microbiome and Cancer: Emerging Trends and Insights
Source: Can J Infect Dis Med Microbiol. 2025 Sep 20;2025:6639127. doi: 10.1155/cjid/6639127 (PMC12575027; doi:10.1155/cjid/6639127)
Supplement: Supporting Information — Additional supporting information can be found online in the Supporting Information section. [file 6639127.f1.docx]

Search quires and refinement procedure:

1: TI = ((oral or salivary or saliva or tongue or dental or periodontal or endodontic or teeth or tooth or *gingiva* or palmatum or palatine or palatal or pharynx or throat or cheek* or tonsil* or "buccal mucosa" or "hard palate" or "soft palate" or "oral cavity" or “palatoglossal arch” ) and (microb* or microflora or microorganism* or micro-organism* or flora or microecolog* or *bacter* or *fung* or mycobio* or mycolog* or *virus* or pathogen* or dysbiosis or Candida or Candida albican* or Porphyromonas gingivalis or Fusobacterium nucleatum or Streptococcus or Helicobacter pylori))

2: TI = (“Tumor*” OR “Tumour*” OR “Cancer*” OR “Neoplasia*” OR “Neoplasm*” OR “Malignanc*” OR “Carcinoma*” OR “Oncolog*” OR “Melanoma*” OR “Lymphoma*” OR “Scrcoma*” OR "Adenocarcinoma*" OR "Leukemia*" OR "Myeloma*" OR "Blastoma*" OR "Glioma*" OR "Carcinogen*" OR "Oncogen*") OR AK = (“Tumor*” OR “Tumour*” OR “Cancer*” OR “Neoplasia*” OR “Neoplasm*” OR “Malignanc*” OR “Carcinoma*” OR “Oncolog*” OR “Melanoma*” OR “Lymphoma*” OR “Scrcoma*” OR "Adenocarcinoma*" OR "Leukemia*" OR "Myeloma*" OR "Blastoma*" OR "Glioma*" OR "Carcinogen*" OR "Oncogen*")

3: #1 AND #2 and Meeting Abstract or Letter or Editorial Material or Correction or News Item or Proceeding Paper or Book Chapters or Retraction or Retracted Publication (Exclude – Document Types) and 2024 or 2023 or 2022 or 2021 or 2020 or 2019 or 2018 or 2017 or 2016 or 2015 or 2014 or 2013 or 2012 or 2011 or 2010 (Publication Years)

Chaining search:

https://webofscience.clarivate.cn/wos/woscc/summary/3a0df30f-6022-4fa1-aa46-6df144174945-014631475b/times-cited-descending/1
